# The effect of generative artificial intelligence literacy on academic achievement: the mediating role of academic self-efficacy and the moderating role of critical thinking

**DOI:** 10.3389/fpsyg.2026.1761562

**Published:** 2026-03-13

**Authors:** Yingchun Wang

**Affiliations:** School of Ethnology and Sociology, Minzu University of China, Beijing, China

**Keywords:** academic achievement, academic self-efficacy, critical thinking, generative artificial intelligence, generative artificial intelligence literacy

## Abstract

The underlying mechanism through generative artificial intelligence (GenAI) literacy impacts academic achievement remains insufficiently examined. This study aims to investigate how GenAI literacy influences academic achievement via the mediating role of academic self-efficacy, and whether critical thinking moderates the relationship between GenAI literacy and academic self-efficacy. A total of 744 university students (382 males, 362 females) completed questionnaires measuring their Gen AI literacy, academic achievement, academic self-efficacy, and critical thinking skills. The results supported the mediating effect of academic self-efficacy, suggesting that GenAI literacy can enhance academic achievement by boosting students’ academic self-efficacy. Furthermore, the relationship between GenAI literacy and academic self-efficacy was found to be moderated by critical thinking. Specifically, the positive effect of GenAI literacy on academic self-efficacy was more pronounced among students with high levels of critical thinking. This research offers insights for developing specific interventions to leverage GenAI literacy, foster academic self-efficacy, and cultivate critical thinking, thereby improving academic outcomes. Limitations of the study and directions for future research are also discussed.

## 1 Introduction

In an era where artificial intelligence (AI), particularly Generative AI (Gen AI), is reshaping the higher education ecosystem at an unprecedented pace, investigating the underlying mechanisms of its impact on student academic development has become an urgent scholarly imperative. On one hand, Gen AI’s capacity for high-quality text generation has ignited a “phenomenal” debate within the field of education (Wang and Li, 2023). GenAI offered significant benefits in STEM education, positively influencing individual learners, teachers, and the learning and teaching process (Zhu et al., 2026). On the other hand, the broader digital transformation process presents formidable challenges to the cultivation of innovative talent, including potential crises of agency for both teachers and students, data security and ethical dilemmas, academic misconduct, and educational inequality (Rasul et al., 2023). However, the effectiveness of Gen AI is not a universal technological boon; its impact is profoundly conditioned by individual psychological characteristics and cognitive abilities. Therefore, amid a deepening technological revolution, the critical question is how to leverage the instrumental utility of Gen AI to advance the digital transformation of higher education and enhance the multifaceted competencies of contemporary university students. Addressing this question is key to fostering the innovative talent required for the future.

There is growing evidence that Gen AI is becoming an integral part of the daily academic and personal lives of university students. For instance, a survey in the United States revealed that 89% of college students use ChatGPT to complete assignments, and 53% leverage it for writing essays. In a similar vein, The Times reported that nearly half of the students at the University of Cambridge use ChatGPT in their studies (Moorhouse et al., 2023). As the use of GenAI tools becomes a prevalent learning approach, it is particularly urgent and critical to explore the mechanisms through which they affect the critical thinking of contemporary university students. Although some researchers have begun to investigate the impact of GenAI on student growth and development—including learning motivation, engagement, and academic performance (Yilmaz and Yilmaz, 2023)—the findings remain inconclusive. Furthermore, there has been insufficient attention on students’ critical thinking, with a notable scarcity of empirical analysis in local contexts. Therefore, this study, grounded in a practical perspective, seeks to determine the pathways through which Gen AI influences academic performance and the conditions under which these pathways may be altered. To address these questions, we propose a moderated mediation model to systematically elucidate the mechanism by which Gen AI literacy affects academic performance, focusing specifically on the mediating role of academic self-efficacy and the moderating effect of critical thinking.

In China, the integration of artificial intelligence into education has evolved from a strategic vision to a multi-layered, widespread practical landscape. At the macro level, this process has been driven by strong national policies providing top-level design and momentum. The State Council’s New Generation Artificial Intelligence Development Plan (2018) explicitly calls for “using intelligent technology to accelerate reforms in talent cultivation models and teaching methods” (State Council of the People’s Republic of China, 2018). Furthermore, China’s Education Modernization 2035 (2019) identifies “accelerating educational transformation in the information age” as a core mission aimed at building an intelligent, personalized educational ecosystem (Ministry of Education of the People’s Republic of China, 2019). These policy initiatives have laid the institutional groundwork for AI to transition from a peripheral tool to a central element in education. Against this backdrop, applications such as adaptive learning systems and intelligent assessments (Chen et al., 2018) have gained initial validation in practice, primarily valued for enhancing instructional efficiency and personalizing learning paths. However, the explosive growth of Gen AI, powered by large language models, is triggering a more profound paradigm shift in the educational domain. Unlike previous AI, which primarily served as “intelligent tutors” or “assessment tools,” GenAI deeply intervenes in the entire student process of knowledge inquiry, text construction, and problem-solving through its powerful content generation and interactive capabilities. Students have spontaneously developed diverse usage strategies—from brainstorming and drafting outlines to seeking code optimization advice. While this autonomy and pervasiveness in technology use demonstrate unprecedented potential for learning empowerment, they have also fueled new academic anxieties. As technology dramatically lowers the barriers to knowledge acquisition and content creation, a key concern is whether students might fall into a “technology dependence” trap, thereby undermining their intrinsic motivation for autonomous learning and their higher-order thinking skills. Some research has already cautioned against over-reliance on generative chatbots, warning that it may lead to “mediocrity, hindering progress and innovation” (Zheng et al., 2024) and suggesting that this convenience could negatively impact the development of critical thinking. Therefore, systematically examining the actual impact of GenAI on academic achievement, and deeply analyzing its underlying psychological mechanisms and boundary conditions, constitutes the core motivation and practical entry point for this study.

## 2 Theoretical foundation and research hypotheses

### 2.1 Theoretical foundation

The theoretical framework of this study is rooted in Albert Bandura’s Social Cognitive Theory (SCT). The central tenet of SCT is the principle of triadic reciprocal determinism, which posits a continuous and dynamic interaction among personal factors (e.g., cognition, affect, and beliefs), behavior, and the environment (Bandura, 1997). By rejecting earlier theories of unidirectional determinism, SCT proposes that these three factors influence one another in a reciprocal fashion, forming a “triadic interactive model” (Cialdini et al., 1999). This theory offers a powerful integrative framework for the current research, enabling the synthesis of seemingly disparate variables—namely, technological literacy, psychological beliefs, cognitive abilities, and academic outcomes—into a coherent model.

Within the context of our research model, SCT’s components are specified as follows. The environment is manifested through the pervasive technological landscape of Gen AI. Students’ engagement with this new digital milieu allows them to develop their GenAI literacy, which serves as a critical external input and skill base for their academic progress. The personal factors, which are central to our investigation, include the student’s internal cognitive and belief systems. Prominently featured among these is academic self-efficacy, a cornerstone concept in SCT that captures a student’s belief in their ability to succeed in academic tasks and functions as a primary driver of academic motivation and behavior. Also included is critical thinking, conceptualized as a higher-order cognitive skill that indicates the sophistication of an individual’s cognitive processing and knowledge construction. Finally, the behavioral factor is represented by academic performance, which is the quantifiable achievement outcome resulting from the dynamic interplay between these personal and environmental determinants.

Specifically, the application of SCT provides theoretical support for our hypotheses in two ways. First, SCT emphasizes the critical role of vicarious experiences and mastery experiences in shaping self-efficacy. When students successfully leverage their Gen AI literacy to navigate AI tools and complete academic tasks, this constitutes a powerful mastery experience. This provides a direct theoretical rationale for the mediating hypothesis (mediation: GenAI Literacy → Academic Self-Efficacy → Academic Performance). Second, SCT posits that personal cognitive factors are crucial in moderating how environmental influences are internalized into individual beliefs. Students with varying levels of critical thinking will decode, evaluate, and internalize information from the AI “environment” in fundamentally different ways. This offers a profound theoretical basis for proposing critical thinking as a moderator that influences the cognitive transformation process encapsulated in the “GenAI Literacy → Academic Self-Efficacy” pathway.

Therefore, the moderated mediation model constructed in this study represents a specific application and empirical test of SCT’s triadic reciprocal determinism within the context of AI in education. It aims to elucidate the complex dynamic process through which technological literacy (a skill from environmental interaction) influences academic outcomes (behavior) by reshaping individual beliefs (personal factors), a pathway that is itself conditioned by the individual’s higher-order cognitive abilities (also personal factors).

### 2.2 Literature review and research hypotheses

#### 2.2.1 Generative artificial intelligence literacy and academic performance

Within the framework of Social Cognitive Theory (SCT), intrinsic motivation and innovative self-efficacy are often used to explain the mechanisms through which social contextual factors influence individual innovative behavior. Numerous studies have confirmed the positive effects of innovative self-efficacy and intrinsic motivation on employee innovation (Gong, 2010; Shin and Zhou, 2003). As a core competency for effective learning, working, and living in the intelligent era, GenAI literacy transcends mere technical operation. It is defined as a comprehensive, multidimensional construct encompassing awareness, use, evaluation, and ethics (Wang et al., 2023), and this study adopts these four dimensions for its investigation. Previous research has indicated that integrating GenAI tools into teaching can foster student development in areas such as academic performance (Anyanwu et al., 2024), problem-solving skills (Lee et al., 2024), and creativity (Habib et al., 2024). Moreover, studies have highlighted that GenAI’s Natural Language Processing (NLP) capabilities can be used to grade student writing and provide explanatory feedback, thereby helping learners continuously improve their reading and writing skills (Chen et al., 2023). In summary, the existing literature suggests a positive relationship between AI literacy and academic performance.

Specifically, at the awareness level, one study found that readiness, interactivity, and ethical awareness significantly influence students’ adoption of AI applications, whereas trust and performance expectations do not have a significant effect (Schmidt et al., 2025). At the use level, students can master the basic operations of GenAI to quickly gather preliminary information, generate text drafts, or debug code. Anyanwu et al. (2024) discovered that applying ChatGPT significantly improves student engagement and learning outcomes in technical education, with students showing marked improvement in core skills such as “knowing, applying, and reasoning”. Furthermore, intelligent simulated learning environments have been shown to significantly enhance students’ performance in concept acquisition and knowledge organization (Du and Gu, 2022), and their human-like conversational format can stimulate creativity and imagination (Zhang, 2022). Therefore, at this level, when students can proficiently leverage AI tools for information retrieval, data analysis, and content drafting, they can optimize the allocation of their cognitive resources to focus on higher-order creative and analytical tasks, thereby improving the efficiency and quality of their academic output (Ouyang et al., 2022). However, research also points to the adverse effects of GenAI on learning. EsCalante et al. (2023) argued that while helpful for understanding concepts and providing information, it may undermine students’ critical thinking and independent learning abilities. Over-reliance on GenAI can hinder the development of problem-solving skills (Habib et al., 2024). Moreover, the premature introduction of these tools may lead to student over-dependence, subsequently impairing their writing skills (WaRschauer et al., 2023) and reducing academic performance in relevant subjects. In more severe cases, it could give rise to ethical risks such as the erosion, degradation, and domestication of human agency (Jin and Li, 2023).

The evaluation dimension acts as a crucial “gatekeeper” for academic quality. While some research suggests that AI can surpass traditional assessment methods in breadth, depth, and objectivity (Zhang and Tang, 2023), its outputs are often plagued by factual errors, logical fallacies, and hidden biases—a phenomenon known as “AI hallucinations.” Students with a high level of evaluation literacy maintain what can be termed “epistemological vigilance.” They critically cross-verify the accuracy of AI-generated information, scrutinize the logic of its arguments, and identify potential biases. This critical capacity directly determines whether a student can transform AI from an unreliable source of information into a rigorously vetted, high-quality knowledge-assistive tool. Research has advanced the field’s understanding by highlighting how AI can bridge the gap between traditional and AI-enhanced assessment methods, particularly in scaffolding both formative and summative evaluation practices (Krumsvik, 2025). Ultimately, this ability to critically evaluate is paramount, as it ensures the rigor and accuracy of academic work and, consequently, exerts a significant influence on academic performance.

The ethics dimension serves as a “firewall” for one’s academic career. Although AI holds immense potential for enhancing learning efficiency and academic performance, its successful implementation hinges on addressing critical issues related to accuracy, cognitive disengagement, and ethical implications. A balanced approach is essential to ensure equitable, effective, and responsible learning experiences in AI-enhanced educational environments (Krumsvik, 2025). Ethical literacy requires students to deeply understand and consciously adhere to norms of academic integrity, clearly distinguishing between AI-assisted work and plagiarism. Furthermore, it encompasses a broader awareness of ethical issues such as data privacy and algorithmic fairness. Students possessing this literacy can leverage the conveniences of technology while avoiding the risks of academic misconduct—such as direct copying or data fabrication—that arise from improper AI use. This not only safeguards their grades on individual assignments but also upholds their long-term academic integrity.

Given the literature mentioned above, we propose the following hypothesis:

*H1*: GenAI literacy, specially awareness (H1a), usage (H1b), evaluation (H1c) and ethics (H1d), would improve academic performance.

#### 2.2.2 The mediating role of academic self-efficacy in the relationship between GenAI literacy and academic performance

The positive influence of AI literacy on academic performance is not direct; instead, its effectiveness is largely actualized by fostering and enhancing students’ intrinsic motivational beliefs. Central to this process is academic self-efficacy, defined as an individual’s judgment of their capability to succeed in academic tasks (Bandura, 1997). This belief serves as a core motivational source driving academic achievement.

First, the development of AI literacy is a significant antecedent to academic self-efficacy. According to Social Cognitive Theory (SCT), “mastery experiences” are the most powerful source for building self-efficacy. Previous research has already highlighted the significant advantages of AIGC in enhancing students’ self-efficacy (Huang et al., 2024). Further, Bai and Wang (2025) indicated that both the quality of GAI interaction and its output positively influence creative self-efficacy. When students successfully leverage GenAI tools to overcome a complex programming challenge, construct a well-structured literature review, or rapidly generate a creative design draft, these successful experiences—embodying a “can-do” attitude—are directly internalized as positive appraisals of their own academic capabilities. Supporting this, existing research has shown that LLM-based Gen AI technologies, such as ChatGPT and other chatbots, significantly impact student learning performance through mechanisms including self-efficacy, fairness and ethics, and creativity (Shahzad et al., 2025). Furthermore, factors such as the tool’s utility, social presence, legitimacy, as well as the enjoyment and motivation it provides, all contribute to a positive attitude towards its use in learning environments (Tiwari et al., 2024). When students can consciously evaluate, identify, and correct errors in AI-generated content, they transition from being passive tool users to active knowledge masters with greater cognitive authority. This sense of control and intellectual superiority significantly enhances their confidence in making academic judgments. Indeed, prior research has established a positive correlation between an individual’s proficiency with a new technology and their level of self-efficacy (Wang and Wu, 2008).

Second, academic self-efficacy acts as a psychological bridge connecting ability beliefs to academic behaviors, and its positive effect on academic performance is well-documented (Zimmerman, 2000; Meng and Zhang, 2023; Wei and Chou, 2020). Research has further explored the mechanisms underlying this relationship. For instance, Komarraju and Nadler (2013) argued that students with low self-efficacy tend to believe that intelligence is innate and unchangeable, whereas those with high self-efficacy pursue mastery goals, which involve tackling challenges and acquiring new knowledge, as well as performance goals, which focus on achieving good grades and outperforming others. Similarly, Wu and Fu (2024) contended that students with high self-efficacy are more inclined to set challenging learning goals; both mastery-approach and performance-approach goals are significantly and positively correlated with academic achievement, making it more likely for these students to attain high grades.

Given the personalized and real-time interactive nature of GAI tools, the interaction between students and GAI is expected to influence their academic performance through the mediating role of self-efficacy (Liang et al., 2023). This heightened confidence, in turn, drives students to engage in their studies with a more positive attitude and greater persistence, ultimately leading to superior academic outcomes. This psychological chain of” Literacy → Belief → Behavior” constitutes the core mediating pathway of our study. Therefore, we propose the following mediation hypothesis:

*H2*: Academic self-efficacy mediates the relationship between AI literacy and academic performance. Specially, academic self-efficacy exerts significant mediating effects among awareness, usage, evaluation and ethics and academic performance, with these hypotheses specified as H2a H2b, H2c and H2d, respectively.

#### 2.2.3 Critical thinking plays a moderating role between artificial intelligence literacy and academic self-efficacy

This study aims to analyze the joint effect of critical thinking and AI literacy on students’ academic self-efficacy, exploring whether a significant interaction exists between human and artificial intelligence. Critical thinking, as a higher-order cognitive ability involving analysis, evaluation, reasoning, and reflection (Ennis, 1985), is defined as “the thought process of identifying issues within a subject, phenomenon, or proposition, while simultaneously formulating a logically-grounded (1993) claim based on one’s own reasoning”. It is this ability that determines the mode and depth of an individual’s interaction with AI-generated information.

Existing research has delineated the distinct characteristics of individuals with high versus low critical thinking skills. This implies that the conversion of AI literacy into academic self-efficacy is not a constant process; rather, a student’s level of critical thinking may function as a crucial “amplifier” or “attenuator” in this relationship. We therefore posit that the level of critical thinking significantly moderates the strength of the impact of AI literacy on academic self-efficacy. This moderation effect can be understood by contrasting the experiences of two student groups.

For students with high critical thinking, they tend to perceive Gen AI (GenAI) not as an oracle, but as a “cognitive scaffold” or an “intellectual sparring partner.” Their engagement with AI is deep and dialectical. They do not merely consume information; instead, they actively and habitually question, validate, and analyze the AI’s outputs, scrutinize its underlying assumptions, and integrate it with their own knowledge frameworks (an evaluative process). This approach aligns with perspectives highlighting unique domains of human cognition, such as flexible attention, robust reasoning in novel contexts, and integrated sense-making, which may be distinct from artificial intelligence (Mogi, 2024). In this dynamic, the AI’s output becomes a catalyst for deeper thought, not a final answer. Each successful “correction” or “surpassing” of the AI serves as a confirmation of their own higher-order thinking abilities, fostering a profound sense of intellectual agency—an experience of “I am more discerning than the machine.” Consequently, the affordances of AI are effectively internalized as an affirmation of their unique cognitive strengths, making the pathway from AI literacy to academic self-efficacy exceptionally direct and potent.

Conversely, for students with low critical thinking, a failure to effectively identify and leverage GenAI in higher education can hinder their academic self-efficacy (Saúde et al., 2024). They are more inclined to treat GenAI as an “all-knowing answer machine” or a “cognitive crutch.” Their interaction pattern is often superficial, characterized by an uncritical “copy-and-paste” usage aimed at quickly obtaining a plausible-looking answer. This can lead to a state of “metacognitive laziness” (Fan et al., 2025). Although this approach may appear to boost short-term task efficiency, any resulting success is often externally attributed to the tool’s power rather than their own competence. This positive attitude toward the tool’s utility may drive their intention to use it, but the engagement remains shallow (Wang et al., 2025). Over time, this dependency may even erode their motivation and capacity for independent thought, fostering a sense of helplessness—a feeling of being “unable to learn without AI.” In this scenario, even if their operational AI skills improve, this enhancement fails to translate effectively, if at all, into robust, intrinsic academic confidence.

Based on this reasoning, we propose the following hypothesis:

*H3*: Critical thinking moderates the relationship between AI literacy (awareness, usage, evaluation and ethics) and academic self-efficacy. Specifically, the positive effect of awareness, usage, evaluation and ethics on academic self-efficacy is stronger for students with high levels of critical thinking than for those with low levels of critical thinking, with these hypotheses specified as H3a H3b, H3c and H3d, respectively.

## 3 Methods

### 3.1 Participants and procedure

A total of 1,000 university students in mainland China (including Beijing, Inner Mongolia, Guangdong, Heilongjiang, Jiangsu, Shandong, Hebei, Fujian, Henan, Zhejiang, Shanghai, Anhui, Hubei, Sichuan, Hunan, Chongqing) completed study questionnaires. After excluding questionnaires due to poor response quality. The final sample included 744 participants aging from 16 to 28 years (*M* = 20.61, *SD* = 1.62), with 382 (51.3%) boys, 362 (48.7%) girls; 118 (15.9%) in the first year, 238 (32.0%) in the second year, and 227 (30.5%) in the third year, and 161 (21.6%) in the fourth year; 428 (57.5%) from urban areas, 316 (42.5%) from rural regions.

Data were collected through Wenjuanxing between September 1st and November 1st, 2025. Information sheet and the link to the survey were sent to potential participants online. Participants indicated their consent by completing the questionnaire. It took up to 10 min to complete the survey.

### 3.2 Measures

#### 3.2.1 Artificial intelligence literacy

Artificial intelligence literacy was measured using the 12-item Artificial Intelligence Literacy scale developed by Wang et al. (2022). The scales include four dimensions, i.e., awareness (e.g., “I can distinguish between smart devices and non-smart devices”), usage (e.g., “I can skillfully use AI applications or products to help me with my daily work”), evaluation (e.g., “I can evaluate the capabilities and limitations of an AI application or product after using it for a while”) and ethics (e.g., “I am always alert to the abuse of AI technology”), which contains 3 items, respectively. Items were rated on a 7-point scale ranging from 1 (extremely disagree) to 7 (extremely agree), with higher average scores on each subscale indicating higher levels of the dimension. Confirmatory factor analysis (CFA) showed that the scale fitted the data well (*χ*^2^/df = 5.01, RMSEA = 0.07, NFI = 0.93, TLI = 0.92, SRMR = 0.05). The Cronbach’s alpha was 0.88.

#### 3.2.2 Academic self-efficacy

Academic self-efficacy was measured using the 8-item self-efficacy subscale of the Motivated Strategies for Learning Questionnaire (MSLQ; Pintrich and De Groot, 1990). This scale has shown good psychometric properties in Chinese samples (e.g., Chen, 2006). Sample items included “I’m certain I can understand the ideas taught in this course”. Items were rated on a 7-point scale ranging from 1 (extremely disagree) to 7 (extremely agree), with higher average scores of the eight items indicating higher levels of academic self-efficacy. Confirmatory factor analysis (CFA) showed that the scale fitted the data well (χ^2^/*df* = 3.49, RMSEA = 0.06, NFI = 0.98, TLI = 0.98, SRMR = 0.02). Cronbach’s alpha in the present study was 0.91.

#### 3.2.3 Critical thinking

Critical thinking was measured using the 17-item Critical Thinking scale developed by Ren (2023). The scales include three dimensions, i.e., critical analysis skills (e.g., “I usually follow predefined steps in problem-solving”), openness to critique (e.g., “I tend to stick to the existing method rather than seeking alternatives”), and application of critical thinking (e.g., “I often brainstorm additional strategies when faced with challenges”), which contains 8, 5, 4items, respectively. Items were rated on a 7-point scale ranging from 1 (extremely disagree) to 7 (extremely agree), with higher average scores on each subscale indicating higher levels of the dimension. Confirmatory factor analysis (CFA) showed that the scale fitted the data well (*χ*^2^/df = 2.25, RMSEA = 0.04, NFI = 0.96, TLI = 0.97, SRMR = 0.04). The Cronbach’s alpha was 0.88.

#### 3.2.4 Academic achievement

Academic achievement was measured using the 6-item academic achievement scale (Wang et al., 2011). Sample items included “The extent to which I complete work tasks in accordance with instructor requirements”. Items were rated on a 6-point scale ranging from 1 (highly unlikely) to 7 (highly likely), with higher average scores of the six items indicating higher levels of academic achievement. Confirmatory factor analysis (CFA) showed that the scale fitted the data well (*χ^2^*/*df* = 2.90, RMSEA = 0.05, NFI = 0.99, TLI = 0.99, SRMR = 0.02). Cronbach’s alpha in the present study was 0.91.

### 3.3 Data analytic approach

Firstly, we used SPSS 24.0 to obtain descriptive statistics and Pearson’s correlation for artificial intelligence literacy, academic self-efficacy, critical thinking, academic achievement. Secondly, we tested mediation model and moderated mediation model with structural equation modeling (SEM) using Amos 24.0. The significance of the indirect and moderated mediation effects was tested by setting the bootstrap number to 5,000 and the bias-corrected confidence interval to 95%. The 95% bias-corrected confidence interval do not contain zero, indicating the indirect and moderated mediation effects were significant (Preacher and Hayes, 2008).

## 4 Results

### 4.1 Descriptive analysis and intercorrelations

Means, standard deviations and Pearson’s correlation of studied variables are presented in Table 1. There were significant and positive associations between awareness, usage, evaluation, ethics, academic self-efficacy, critical thinking and academic achievement (all *p* < 0.001).

**Table 1:** Descriptive statistics and intercorrelations of variables of interest.

Variable	*M*	SD	1	2	3	4	5	6	7
1. Awareness	4.69	1.00	1						
2. Usage	4.69	1.04	0.68^***^	1					
3. Evaluation	4.63	1.13	0.64^***^	0.65^***^	1				
4. Ethics	4.67	1.05	0.55^***^	0.62^***^	0.56^***^	1			
5. Academic self-efficacy	4.58	1.12	0.40^***^	0.39^***^	0.38^***^	0.42^***^	1		
6. Critical thinking	4.42	0.87	0.51^***^	0.49^***^	0.47^***^	0.50^***^	0.23^***^	1	
7. Academic achievement	3.98	1.20	0.45^***^	0.44^***^	0.41^***^	0.46^***^	0.42^***^	0.33^***^	1

^***^p < 0.001.

### 4.2 The mediating effect of academic self-efficacy on the relation between artificial intelligence literacy and academic achievement

After standardizing all the variables in the model, Model 4 in Process 3.3 showed that the total effects of awareness [*β* = 0.45, SE = 0.03, *p* < 0.001, (0.39, 0.51)], usage [*β* = 0.44, SE = 0.03, *p* < 0.001, (0.38, 0.51)], evaluation [*β* = 0.41, SE = 0.03, *p* < 0.001, (0.23, 0.36)], ethics [*β* = 0.46, SE = 0.03, *p* < 0.001, (0.39, 0.52)]on academic achievement were significant. The direct effect of awareness [*β* = 0.33, SE = 0.03, *p* < 0.001, (0.27, 0.40)], usage [*β* = 0.33, SE = 0.03, *p* < 0.001, (0.26, 0.39)], ethics [*β* = 0.30, SE = 0.03, *p* < 0.001, (0.23, 0.36)] and evaluation [*β* = 0.34, SE = 0.03, *p* < 0.001, (0.27, 0.41)] on academic achievement were also significant, indicating awareness, usage, evaluation and ethics can improve the academic achievement of university students. H1a, H1b, H1c and H1d were thus verified.

The results also showed that the effect of awareness [*β* = 0.40, SE = 0.03, *p* < 0.001, (0.33, 0.47)], usage [*β* = 0.39, SE = 0.03, *p* < 0.001, (0.33, 0.46)], evaluation [*β* = 0.38, SE = 0.03, *p* < 0.001, (0.32, 0.45)] and ethics [*β* = 0.42, SE = 0.03, *p* < 0.001, (0.36, 0.49)] on academic self-efficacy were also significant, and academic self-efficacy positively significantly predicted academic achievement [*β* = 0.29, SE = 0.03, *p* < 0.001, (0.22, 0.36); *β* = 0.29, SE = 0.03, *p* < 0.001, (0.23, 0.36); *β* = 0.31, SE = 0.03, *p* < 0.001, (0.24, 0.38); *β* = 0.28, SE = 0.03, *p* < 0.001, (0.21, 0.35)] when independent variable are awareness, usage, evaluation and ethics. Moreover, the 95% confidence intervals for the indirect effect of awareness [0.08, 0.15], usage[0.08, 0.15], evaluation [0.09, 0.16], ethics [0.08, 0.15] on academic achievement through academic self-efficacy did not include zero, further indicating the presence of a significant mediating relationship, indicating H2a H2b, H2c and H2d were thus verified. Mediation effects accounted for 25.78, 25.70, 28.73, and 25.57% of the total effect, respectively (see Table 2).

**Table 2:** Relationship between artificial intelligence literacy and academic achievement: mediating effects.

Relationship	Path	*β*	SE	Bias-corrected CI (95%)		*t*
				Lower bound	Upper bound	
Awareness → academic achievement	Total effect	0.45	0.03	0.39	0.51	13.72^***^
	Direct effect	0.33	0.03	0.27	0.40	9.79^***^
	Awareness → academic self-efficacy	0.40	0.03	0.33	0.47	11.88^***^
	Academic self-efficacy → academic achievement	0.29	0.03	0.22	0.36	8.42^***^
Usage → academic achievement	Total effect	0.44	0.03	0.38	0.51	13.46^***^
	Direct effect	0.33	0.03	0.26	0.39	9.59^***^
	Usage → academic self-efficacy	0.39	0.03	0.33	0.46	11.67^***^
	Academic self-efficacy → academic achievement	0.29	0.03	0.23	0.36	8.56^***^
Evaluation → academic achievement	Total effect	0.41	0.03	0.35	0.48	12.36^***^
	Direct effect	0.30	0.03	0.23	0.36	8.58^***^
	Evaluation → academic self-efficacy	0.38	0.03	0.32	0.45	11.29^***^
	Academic self-efficacy → academic achievement	0.31	0.03	0.24	0.38	8.97^***^
Ethics → academic achievement	Total effect	0.46	0.03	0.39	0.52	14.01^***^
	Direct effect	0.34	0.03	0.27	0.41	9.84^***^
	Ethics → academic self-efficacy	0.42	0.03	0.36	0.49	12.65^***^
	Academic self-efficacy → academic achievement	0.28	0.03	0.21	0.35	8.07^***^

^***^p < 0.001.

### 4.3 The moderating effect of critical thinking on the relation between artificial intelligence literacy and academic self-efficacy

Finally, Model 7 in the Process macro and the bias-corrected percentile bootstrap method (sampling times 5,000) were used to determine the significance of the moderating effects. The results showed that the interaction term of awareness and critical thinking (*β* = 0.21, t = 5.98, *p* < 0.001, [0.14, 0.27]), usage and critical thinking was significant [*β* = 0.16, *t* = 4.56, *p* < 0.001, (0.09, 0.23)], evaluation and critical thinking was significant [*β* = 0.20, *t* = 5.97, *p* < 0.001, (0.13, 0.26)], ethics and critical thinking was significant [*β* = 0.18, *t* = 4.95, *p* < 0.001, (0.11, 0.25)] were significant. That is, critical thinking moderated the degree of awareness, usage, evaluation and ethics on academic self-efficacy (Table 3), indicating H3a H3b, H3c and H3d were thus verified.

**Table 3:** Relationship between artificial intelligence literacy and academic achievement: moderated mediating effects.

Predictor	Equation 1: academic self-efficacy			Equation 2: academic achievement		
	*β*	SE	*t*	*β*	SE	*t*
Awareness	0.30	0.04	7.20^***^	0.33	0.03	9.79^***^
Academic self-efficacy				0.29	0.03	8.42^***^
Critical thinking	0.04	0.04	1.09			
Awareness × critical thinking	0.21	0.03	5.98^***^			
usage	0.31	0.04	7.62^***^	0.33	0.03	9.59^***^
Academic self-efficacy				0.29	0.03	8.56^***^
Critical thinking	0.04	0.04	1.10			
Usage × critical thinking	0.16	0.03	4.56^***^			
Evaluation	0.31	0.04	8.02^***^	0.30	0.03	8.58^***^
Academic self-efficacy				0.31	0.03	8.98^***^
Critical thinking	0.05	0.04	1.25			
Evaluation × critical thinking	0.20	0.03	5.97^***^			
Ethics	0.33	0.04	8.10^***^	0.34	0.03	9.84^***^
Academic self-efficacy				0.28	0.03	8.07^***^
Critical thinking	0.03	0.04	0.88			
Ethics × critical thinking	0.18	0.04	4.95^***^			

^***^p < 0.001.

**Figure 1:**
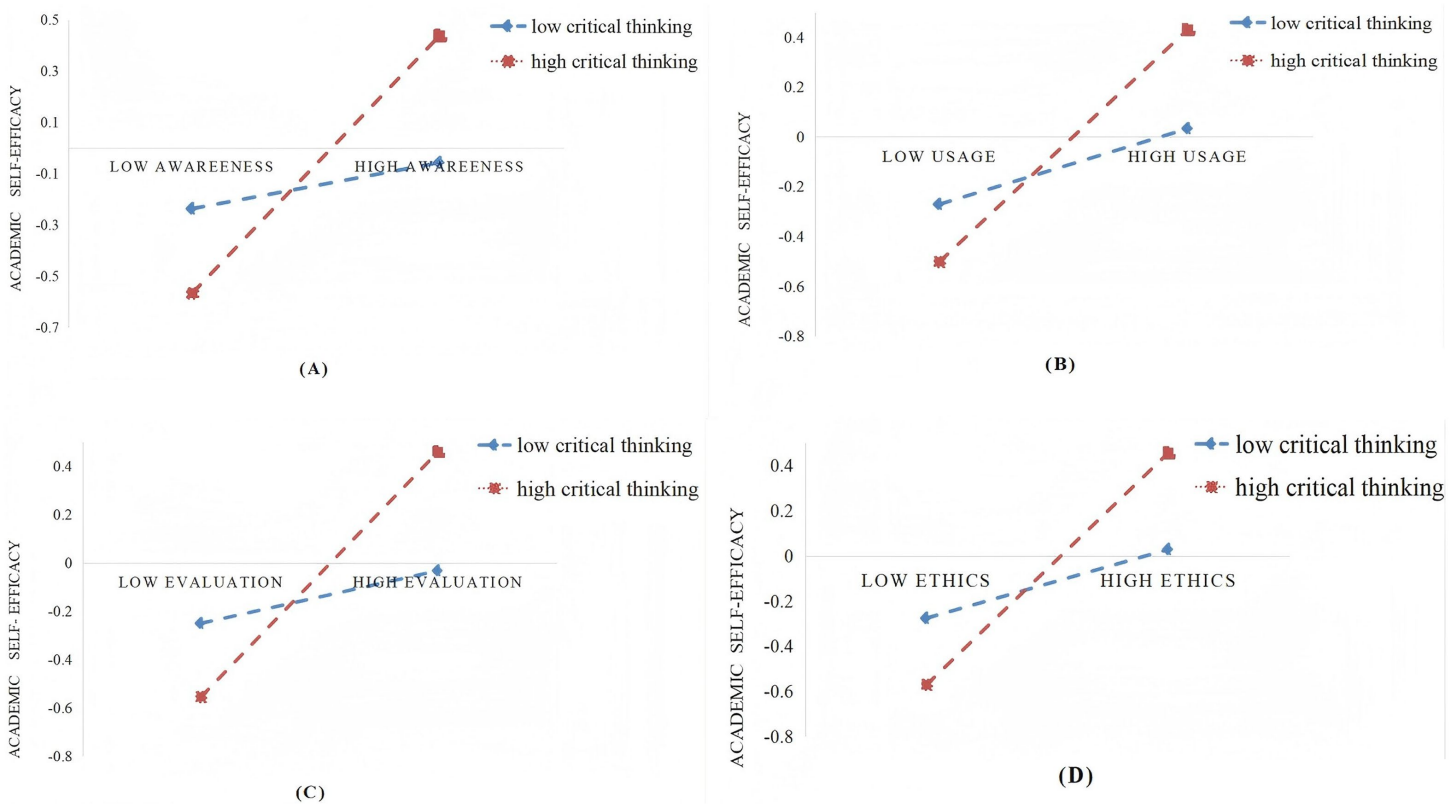
**(A)** Relationship between awareness and academic self-efficacy: moderating role of critical thinking. **(B)** Relationship between usage and academic self-efficacy: moderating role of critical thinking. **(C)** Relationship between evaluation and academic self-efficacy: moderating role of critical thinking. **(D)** Relationship between ethics and academic self-efficacy: moderating role of critical thinking.

To elucidate the moderating effect of critical thinking on the relationship between awareness, usage, evaluation, ethics and academic self-efficacy, we operationalized low and high levels of critical thinking as being one standard deviation below and above the mean. As shown in Figure 1, the relation between awareness and academic self-efficacy was stronger in adolescents who perceived high critical thinking [*β*simple = 0.50, SE = 0.04, *p* < 0.001, (0.42, 0.59)] than college students who perceived the low critical thinking [*β* = 0.09, SE = 0.06, *p* > 0.05, (−0.03, 0.21)]; the relation between usage and academic self-efficacy was stronger in adolescents who perceived high critical thinking [*β*simple = 0.47, SE = 0.04, *p* < 0.001, (0.38, 0.55)] than college students who perceived the low critical thinking [*β* = 0.15, SE = 0.06, *p* < 0.05, (0.03, 0.27)]; the relation between evaluation and academic self-efficacy was stronger in adolescents who perceived high critical thinking [*β*simple = 0.51, SE = 0.05, *p* < 0.001, (0.42, 0.60)] than college students who perceived the low critical thinking [*β* = 0.11, SE = 0.06, *p* = 0.05, (−0.001, 0.22)]; the relation between ethics and academic self-efficacy was stronger in adolescents who perceived high critical thinking [*β*simple = 0.51, SE = 0.04, *p* < 0.001, (0.43, 0.60)] than college students who perceived the low critical thinking [*β* = 0.15, SE = 0.06, *p* < 0.05, (0.03, 0.28)].

## 5 Discussion

Employing a structural equation modeling (SEM) approach, this study systematically investigated the mechanisms through which AI use impacts academic performance. Specifically, it examined the mediating role of academic self-efficacy and the moderating role of critical thinking in this relationship.

### 5.1 The mediating role of academic self-efficacy between AI literacy and academic performance

This study confirms that academic self-efficacy plays a significant mediating role between AI literacy and academic achievement. This finding not only aligns with the fundamental tenets of social cognitive theory but also concurs with recent related research (Bećirović et al., 2025; Yin et al., 2025). Its underlying mechanism can be analogized to the process by which intrinsic motivation enhances innovative performance by fostering interest, increasing engagement, and promoting persistence (Ryan and Deci, 2000). These findings further clarify that the positive impact of AI literacy on academic achievement is not direct but indirect, mediated through the systematic development and enhancement of students’ academic self-efficacy. Specifically, this mediating pathway is realized through the combined effects of AI literacy’s four dimensions:

First, AI awareness serves as the starting point. Guiding students to rationally understand the capabilities, applicable contexts, and limitations of Gen AI helps eliminate unfamiliarity and uncertainty about the technology, reducing anxiety during its use. This enables students to perceive AI as a manageable learning tool rather than an uncontrollable external threat. Such clear cognition itself constitutes psychological empowerment, establishing preliminary confidence for subsequent technology application.

Second, AI application serves as the critical practical component. Effectively utilizing Gen AI to accomplish specific academic tasks—such as literature review, data analysis, programming debugging, or creative generation—enables students to continuously accumulate “mastery experiences.” Each instance of successfully solving an academic problem with the technology directly reinforces their belief in their own competence. The efficiency gains and visible outcomes delivered by the technology significantly reduce learning frustration, thereby positively influencing students’ expectations of their own academic capabilities.

Third, AI evaluation plays a deepening role. When students not only produce content using AI but also critically examine, filter, and optimize its outputs, they transition from passive technology users to active knowledge constructors and quality assessors. This ability to regulate and judge technological outputs powerfully enhances students’ autonomy and sense of control in academic activities. Compared to mere operational skills, the belief that “I can evaluate and improve AI outputs” fosters deeper academic confidence—essentially affirming one’s status as a cognitive agent.

Finally, AI ethics safeguards the sustained development of self-efficacy. Conscientious adherence to ethical norms like academic integrity and data privacy enables students to fully explore technology’s potential within compliant boundaries, thereby reducing psychological burdens and moral concerns stemming from improper use. A clear ethical framework fosters a state of secure, focused technology use. This positive psychological state itself constitutes a vital resource for sustaining and enhancing academic self-efficacy.

In summary, these four interconnected dimensions of AI literacy collectively form a cognitive-behavioral support system that promotes the development of students’ academic self-efficacy. Technology itself does not directly determine academic achievement. Rather, it transforms technological advantages into tangible academic performance by cultivating and reinforcing students’ internal belief that “I can do it.” This finding suggests that AI education should transcend mere skill training, focusing instead on guiding students to progressively build robust academic confidence and self-directed learning abilities through deep, critical, and ethically grounded practice.

### 5.2 The moderating role of critical thinking in the relationship between AI literacy and academic self-efficacy

Another significant finding of this study is that critical thinking plays a notable moderating role in the pathway from artificial intelligence literacy to academic self-efficacy. This conclusion aligns with related research (Ruiz-Rojas et al., 2024; Alqarni, 2025). This indicates that the process of transforming AI literacy into academic self-efficacy is neither linear nor universal. Instead, this conversion efficiency is significantly influenced by individual students’ critical thinking levels. Students with high critical thinking appear to possess a “cognitive amplifier” that enhances the positive psychological effects of AI literacy, while those with low critical thinking may face risks of “efficacy discounting” or even “efficacy backlash.” This moderating effect can be further analyzed through the four dimensions of AI literacy:

At the cognitive level, students with high critical thinking not only recognize AI’s capabilities but also possess deep insights into its underlying mechanisms (e.g., probabilistic text generation) and inherent limitations (e.g., “hallucination” phenomena). This discerning cognitive stance enables them to develop more objective and rational perceptions of AI, avoiding both blind worship and irrational fear. The resulting AI usage expectations become more realistic, laying a solid foundation for developing robust and positive self-efficacy.

This moderating effect is particularly pronounced at the AI application level. Research indicates that when GAl is combined with critical thinking, it promotes deep learning, thereby reducing reliance on prior knowledge. Educators should prioritize cultivating critical thinking to fully leverage the benefits of GAl. These findings underscore the necessity for educational design to balance GAI’s cognitive support with the cultivation of metacognitive skills (Zhao et al., 2025). Conversely, students with weaker critical thinking skills are more prone to falling into a superficial “command-and-copy” mode. For them, the more powerful the technology, the more severe the phenomenon of “cognitive disengagement” becomes. This hinders their ability to gain a genuine sense of competency growth from technology application, naturally constraining the enhancement of their self-efficacy.

The AI assessment dimension itself directly embodies critical thinking within AI application scenarios, forming a powerful virtuous cycle between the two. Research indicates that students with stronger self-regulated learning abilities are more likely to utilize Gen AI for reflective thinking and achieve better development in critical thinking skills. Students with strong intrinsic learning motivation are more likely to leverage Gen AI for reflective thinking and achieve better development in critical thinking skills (Zhang and Liu, 2025).

Students with strong critical thinking abilities often exhibit more developed evaluation dimensions within their AI literacy—actively fact-checking, logically scrutinizing, and value-judging AI-generated outputs. This process of verification and refinement inherently constitutes intensive cognitive training. Successfully identifying and correcting AI fallacies not only prevents information misuse but also fosters cognitive mastery over machine intelligence, significantly enhancing academic self-efficacy.

Within the ethical dimension of AI, critical thinking propels students beyond superficial rule-following into deep ethical reasoning. They reflect on complex issues such as potential biases in AI content, the legitimacy of data sources, and intellectual property attribution. This conscious ethical practice ensures students use artificial intelligence grounded in self-regulated moral foundations, fostering a more stable and secure psychological state. This inner peace, rooted in ethical conviction, effectively counters the erosion of academic self-efficacy by potential ethical anxieties, empowering students to employ AI with greater confidence and responsibility. While acknowledging AI’s advantages in speed and efficiency, students express concerns about its ethical implications and potential impact on critical thinking. Key ethical issues include maintaining academic integrity, ensuring manual verification of AI-generated content, and avoiding overreliance (Pratiwi et al., 2025).

In summary, critical thinking acts as a “core regulatory mechanism” governing the transformation of AI literacy into academic efficacy. It determines whether students become the “intelligent masters” or the “lazy servants” of AI. This finding strongly indicates that when advancing AI education, the cultivation of critical thinking must be placed at an equal or even higher strategic priority. Without corresponding higher-order thinking skills, mere technological empowerment is likely to yield diminishing returns or even backfire, failing to genuinely foster students’ intrinsic academic confidence and ultimate academic achievement.

## 6 Implications and limitations

The empirical findings of this study provide insights with both practical guidance for higher education transformation in the context of generative artificial intelligence. First, educational philosophy must undergo a strategic shift from “risk avoidance” to “competency empowerment,” while precisely defining the utility boundaries for GenAI usage. Currently, GenAI integration in higher education remains unevenly developed. While some institutions have issued guidelines, many others remain undecided in policy formulation (Erhardt et al., 2025). This inconsistency amplifies educators’ widespread concerns about risks such as cheating, misinformation, and privacy breaches (Cervantes et al., 2024). Therefore, educational interventions should not aim to simply encourage or prohibit GenAI use, but rather guide students into and sustain them within the “optimal utility zone.” This challenges the traditional regulatory mindset that views GenAI as a purely academic risk source, advocating instead for its repositioning as a powerful cognitive enhancement tool. Consequently, building a multi-tiered, progressive AI literacy cultivation system is imperative and has become a consensus for addressing GenAI’s opportunities and challenges (Maxwell et al., 2025). A concrete intervention framework could be designed as follows: (1) Foundational Awareness Module: Offer general education courses for all students covering GenAI principles, ethical norms, and academic integrity, addressing the urgent need for ethical guidance from the student perspective (Zhou, 2024). (2) Discipline-Integrated Module: Embed AI application workshops within various disciplines. For instance, computer science courses could explore AI’s utility in programming and learning support (Agbo et al., 2025), while other majors could learn to leverage AI for literature reviews and data analysis, achieving deep integration of AI skills with professional competencies. (3) Advanced Development Module: Through specialized seminars, train students in advanced capabilities like sophisticated prompt engineering and multi-model cross-validation. Teach them to leverage synergistic and conflicting effects within AI systems to enhance higher-order thinking, thereby internalizing the core principle of responsible AI use (Cheung et al., 2026).

Second, instructional design should strategically leverage the cultivation of students’ academic self-efficacy to create positive human-machine collaborative learning experiences. This study confirms that academic self-efficacy plays a crucial mediating role in the conversion of AI literacy into academic achievement. This implies that merely mastering AI skills is insufficient to guarantee academic success; students’ confidence and intrinsic motivation are more critical. Therefore, instructional interventions should shift their focus to leveraging AI as a catalyst for helping students accumulate “mastery experiences.” An effective strategy is ensuring “Task-Technology Fit”—guiding students to apply AI in appropriate tasks to generate positive emotions through cognitive evaluation and enhance learning efficiency (Zhu et al., 2025). For instance, educators can design “stepwise AI-assisted projects”: during the initial essay drafting phase, guide students to use AI for topic ideation (low cognitive load success experience); in the middle phase, employ AI to draft outlines and conduct critical revisions (moderate cognitive load sense of control); in the final phase, emphasize independent completion of core arguments while using AI solely for language polishing (high-level autonomous learning sense of accomplishment). This progressive sequence of successes effectively builds academic confidence. Simultaneously, to promote responsible application, educators should cultivate a GenAI-friendly environment (Han et al., 2025). By establishing AI learning communities,” students gain “vicarious experiences” and “social persuasion” through sharing techniques and collaboratively analyzing AI fallacies, thereby internalizing external technological empowerment into enduring learning motivation. Moreover, research indicates that students achieve superior learning outcomes through text-based interactions with GenAI compared to other media (Liu et al., 2025). This suggests that text-based conversational models should be prioritized when designing interactive experiences like personalized feedback (Tremblay et al., 2025).

Finally, critical thinking training must be deeply integrated as a core element within the complete human-machine collaborative learning loop. This study reveals that critical thinking is not only a core competency required in the GenAI era but also acts as a significant “amplifier of efficacy” in the impact of AI literacy on academic achievement. As emphasized by related research, students’ personal abilities—such as critical thinking—are pivotal to maximizing their benefits in the GenAI era (Qi et al., 2025). Therefore, higher education must develop meticulous teaching strategies to foster ethical GenAI literacy and uphold academic integrity (Jha and Atif, 2025). Specific interventions should not be limited to isolated logical training but should instead establish a teaching model of “AI-assisted generation—critical evaluation—knowledge re-creation.” Under this model, after using AI to generate preliminary content, students must submit a “Critical Analysis Report on AI-Generated Content” based on an evaluation framework covering factual accuracy, argumentative logic, potential biases, and source reliability. As researchers advocate leveraging emerging technologies to cultivate learners’ 21st-century core competencies (Jiang et al., 2025), this process effectively transforms GenAI into a training ground for critical thinking. Subsequently, students must integrate personal insights to complete knowledge “recreation,” clearly distinguishing AI contributions from their original contributions. This closed-loop design aims to drive a fundamental role shift from passive “information recipients” to active “knowledge constructors.” Through intellectual ‘negotiation’ and “interplay” with AI, students achieve a leap in their higher-order thinking abilities.

While this study offers valuable insights, it is subject to several limitations that warrant attention in future research. First, this study employed a cross-sectional design, collecting data on all variables at a single point in time, which inherently limited in its capacity to infer causality. Future research should adopt longitudinal or experimental intervention designs to more definitively establish the temporal sequence and causal effects among the variables. Second, despite the good reliability and validity of the scales used, the self-report method is susceptible to social desirability bias and participants’ subjective perceptions. Future studies could incorporate more objective measures to triangulate the findings. For instance, student academic performance could be measured using a combination of actual GPAs, expert ratings of coursework, or online behavioral data obtained through learning analytics. Third, the scope of moderating variables in this study was focused on critical thinking as a cognitive factor. However, the individual traits influencing the effective use of AI are likely multifaceted. For example, factors such as students’ motivation types (e.g., intrinsic vs. extrinsic), metacognitive abilities, and even personality traits (e.g., Conscientiousness, Openness) could play significant roles in the conversion of AI literacy into academic achievement. Future research could construct more comprehensive models that incorporate a broader range of potential moderating or mediating variables to achieve a more holistic understanding of the underlying mechanisms.

## 7 Conclusion

This study investigated the impact of AI literacy on the academic performance of university students, examining the mediating role of academic self-efficacy and the moderating role of critical thinking on the relationship between AI literacy and academic self-efficacy. The findings not only corroborate previous research in this field but also offer novel insights. A key discovery is that the positive effect of AI literacy on academic achievement is significantly more pronounced for individuals with higher levels of critical thinking. Based on these findings, the study proposes several practical measures aimed at enhancing AI literacy, bolstering academic self-efficacy, and cultivating critical thinking skills.

## Data Availability

The raw data supporting the conclusions of this article will be made available by the authors, without undue reservation.
